# Notch Signalling in Breast Development and Cancer

**DOI:** 10.3389/fcell.2021.692173

**Published:** 2021-07-06

**Authors:** Abigail Edwards, Keith Brennan

**Affiliations:** Division of Cancer Sciences, Faculty of Biology, Medicine and Health, Manchester Academic Health Science Centre, University of Manchester, Manchester, United Kingdom

**Keywords:** Notch signalling, mammary gland development, breast cancer, cancer hallmarks, breast cancer therapy

## Abstract

The Notch signalling pathway is a highly conserved developmental signalling pathway, with vital roles in determining cell fate during embryonic development and tissue homeostasis. Aberrant Notch signalling has been implicated in many disease pathologies, including cancer. In this review, we will outline the mechanism and regulation of the Notch signalling pathway. We will also outline the role Notch signalling plays in normal mammary gland development and how Notch signalling is implicated in breast cancer tumorigenesis and progression. We will cover how Notch signalling controls several different hallmarks of cancer within epithelial cells with sections focussed on its roles in proliferation, apoptosis, invasion, and metastasis. We will provide evidence for Notch signalling in the breast cancer stem cell phenotype, which also has implications for therapy resistance and disease relapse in breast cancer patients. Finally, we will summarise the developments in therapeutic targeting of Notch signalling, and the pros and cons of this approach for the treatment of breast cancer.

## Introduction

At the turn of millennium, there was growing interest in the role Notch signalling played in tissue homeostasis and the aetiology of human diseases. Over the previous decade, all four Notch homologues had been identified in mammals, along with the five Notch ligands. The generation of genetic knockouts in mice had demonstrated the importance of Notch signalling in embryonic development and the aetiology of several human genetic disorders, including Alagille syndrome. There was also growing evidence that aberrant Notch signalling was linked to several different cancers, in particular certain leukaemias. Amongst solid cancers, breast cancer was of particular interest. Integration of the Mouse Mammary Tumour Virus (MMTV) into the Notch1 or Notch4 loci, leading to the expression of an activated form of the respective Notch proteins, had been shown to disrupt mammary gland development and cause tumour development. However, it was unclear whether Notch signalling played a role in the normal development of mammary gland or in the aetiology of breast cancer in humans. Work since, has provided conclusive evidence for both.

## Notch Signalling

**FIGURE 1:**
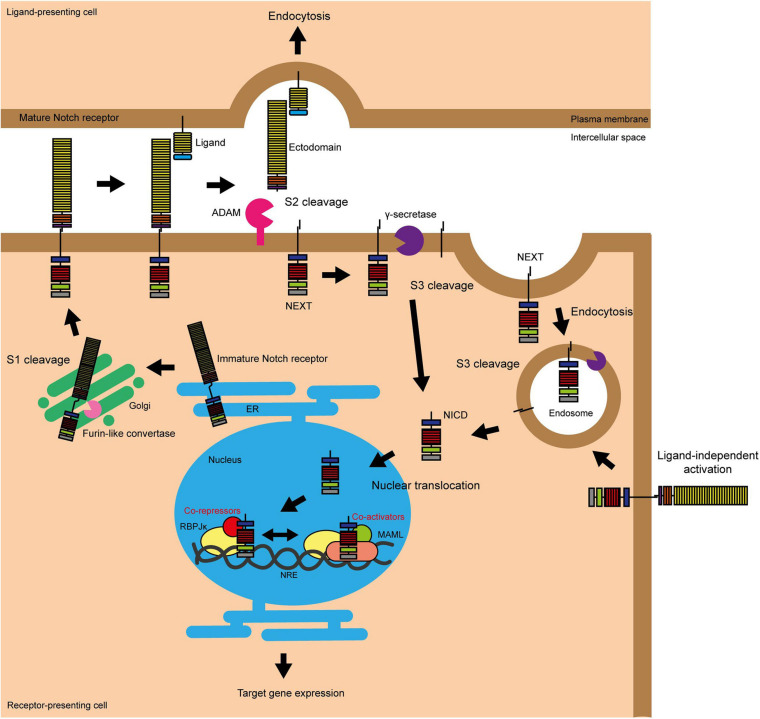
Activation of Notch signalling. The Notch pathway can be activated in two ways, either by interacting with a ligand on an adjacent cell or following endocytosis driven by Deltex. S1 cleavage occurs in the Golgi and mediates the production of the mature Notch heterodimer which is presented on the surface of the cell. Ligand binding stimulates S2 cleavage, which causes the release of the Notch ectodomain and subsequent endocytosis by the ligand-presenting cell. S2 cleavage provides the substrate for γ-secretase, which carries out the final S3 cleavage and releases NICD into the cytoplasm where it can translocate into the nucleus to activate target gene transcription (Fortini, 2009; Aster et al., 2017). The endocytosis of the Notch protein to the multivesicular body driven by Deltex also provides a substrate for γ-secretase and thus target gene transcription (Steinbuck and Winandy, 2018).

At first glance, the Notch signalling pathway is a simple one, with a relatively small number of core signalling components compared to other vital developmental pathways, and lacking in any second messengers, phosphorylation, or amplification steps (Bray, 2006, 2016; Hori et al., 2013; Figure 1). Signalling through the pathway is typically thought to be initiated by physical association between a Notch receptor expressed on the surface of the signal-receiving cell, and a Notch ligand expressed on the surface of the signal-sending cell. However, there is clear evidence that signalling through the pathway can also be initiated in a ligand independent-manner following Deltex-mediated endocytosis localising the Notch protein to the outer surface of the multivesicular body (Steinbuck and Winandy, 2018).

There are four Notch receptors (Notch 1–4). As these receptors are produced in the endoplasmic reticulum (ER), they undergo vital post-translational modification and processing steps in the Golgi. This includes proteolytic cleavage by Furin-like convertase and *O*-glycosylation by protein *O*-fucosyltransferase 1 (POFUT1), protein *O*-glucosyltransferase 1 (POGLUT1) and the Fringe proteins. Furin processing of the Notch receptor is known as S1 cleavage, and results in the presentation of the receptor at the plasma membrane as a heterodimer, with the two fragments linked by non-covalent Ca^2+^ salt bridge interactions (Logeat et al., 1998; Rand et al., 2000). *O*-Glycosylation alters the folding of the Notch protein, increasing its stability and presentation at the cell surface, and changing its interaction with the five Notch ligands, Delta-like 1, 3, and 4 (DLL1, DLL3, and DLL4) and Jagged 1 and 2 (JAG1 and JAG2) (Harvey and Haltiwanger, 2018). Fringe modification of the *O*-glycosylation chains favours binding by the DLL ligands.

Notch receptor-ligand binding triggers endocytosis of the ligand by the ligand-presenting cell (Parks et al., 2000). This induces a mechanical force across the receptor which causes the unfolding of the NRR domain, exposing the S2 site to proteolytic cleavage by disintegrin and metalloprotease (ADAM) proteases (Brou et al., 2000; Nichols et al., 2007). ADAM protease activity causes the release of the Notch ectodomain, leaving the activated and membrane bound form of Notch known as NEXT (Notch extracellular truncation) (Andersson et al., 2011). The Notch ectodomain is endocytosed by the ligand-presenting cell (Hori et al., 2013). NEXT is the substrate for γ-secretase, a complex comprising presenilin, nicastrin, presenilin enhancer 2 (PEN2) and anterior pharynx-defective 1 (APH1), which carries out the third and final Notch proteolytic cleavage (S3) to release NICD into the cytoplasm (Schroeter et al., 1998; De Strooper et al., 1999; Bray, 2006). S3 cleavage by γ-secretase can occur at the plasma membrane or within endosomes as part of NEXT endosomal trafficking (Vaccari et al., 2008; Andersson et al., 2011). Once in the cytoplasm, NICD is transported to the nucleus via importin-α proteins, where it is able to induce target gene transcription (Huenniger et al., 2010).

As well as the ligand-activated Notch signalling pathway outlined above, evidence shows that the core Notch pathway can be activated in a ligand-independent manner through the activity of the E3 ubiquitin ligase Deltex (DTX). In this scenario, full length Notch is trafficked into the cell through endocytosis. DTX functions to stabilise Notch in the endocytic compartment via ubiquitination, and assists in the delivery of the receptor to the limiting membrane of the multivesicular body. Here the receptor undergoes S3 cleavage and NICD is released into the cytoplasm (Shimizu et al., 2014; Steinbuck and Winandy, 2018). There are also suggestions that Notch/DLL and Notch/JAG signalling are distinct, with Notch/DLL signalling favouring the classical lateral inhibition patterning causing cells within a sheet to adopt two different fates in a salt and pepper pattern and Notch/JAG signalling favouring lateral induction causing a group of neighbouring cells to adopt the same fate (Bocci et al., 2020).

In the absence of NICD, Notch target gene expression is repressed by the transcription factor RBPJκ (also known as CBF1) and its co-repressors (Jarriault et al., 1995). These co-repressors, such as RBPJκ-interacting and tubulin-associated (RITA) and silencing mediator for retinoid or thyroid hormone receptors (SMRT)/histone deacetylase (HDAC) 1-associated repressor protein (known as SHARP), compete for NICD binding, as well as actively silencing target gene transcription (Kao et al., 1998; Oswald et al., 2005; Wacker et al., 2011). In the presence of NICD, the co-repressors are displaced and a transcriptional activator complex is formed containing NICD, RBPJκ, and various co-activators including the Mastermind-like (MAML) proteins (Wu et al., 2000; Nam et al., 2006; Wilson and Kovall, 2006). The transcriptional activator complex binds to Notch regulatory elements (NREs) located in gene enhancer elements, resulting in Notch target gene expression (Aster et al., 2017). This is the traditional “switch” model of Notch target gene regulation; however more recent studies have suggested a more dynamic role for RBPJκ than previously thought, involving the movement of the whole transcriptional activator/repressor complex on and off the NRE (Kao et al., 1998; Castel et al., 2013; Bray, 2016).

The classical Notch target genes are the hairy and enhancer of split-related genes; belonging to the HES and HEY families. Hes/Hey proteins are basic helix-loop-helix (bHLH) transcription factors which play key roles during embryonic development as transcriptional repressors. Other canonical Notch target genes include the transcription factors c-Myc, GATA2/3 and Snail; cell cycle regulators E2F, cyclin D1/3, and p21; immune components interleukin 2 receptor subunit alpha [IL2RA (CD25)], pre-T cell receptor α (pTa) and NFκB2; developmental homeobox (HOX) A genes; the matrix metalloprotease ADAM19, and the receptor tyrosine kinase platelet-derived growth factor receptor beta (PDGFRβ) (Oswald et al., 1998; Deftos et al., 2000; Rangarajan et al., 2001; Ronchini and Capobianco, 2001; Reizis and Leder, 2002; Robert-Moreno et al., 2005; Maillard et al., 2006; Palomero et al., 2006; Weerkamp et al., 2006; Weng et al., 2006; Amsen et al., 2007; Fang et al., 2007; Jin et al., 2008; Sahlgren et al., 2008; Borggrefe and Oswald, 2009; Joshi et al., 2009; Bivik et al., 2016). Finally, like many key signalling pathways, Notch is involved in crosstalk with other notable signalling networks in the regulation of development, inflammation and cell function. This is particularly important to consider in the context of Notch signalling in oncogenesis and the design of Notch-targeting therapeutic approaches. For example, Notch interacts with the Wnt, NFκB, TGFβ, HIF1α, YAP/TAZ, EGFR and Akt signalling pathways (Andersson et al., 2011; Bray, 2016; Fazio and Ricciardiello, 2016; Siebel and Lendahl, 2017; Totaro et al., 2018).

## Mammary Stem Cells, Progenitors and Lineage Determination

**FIGURE 2:**
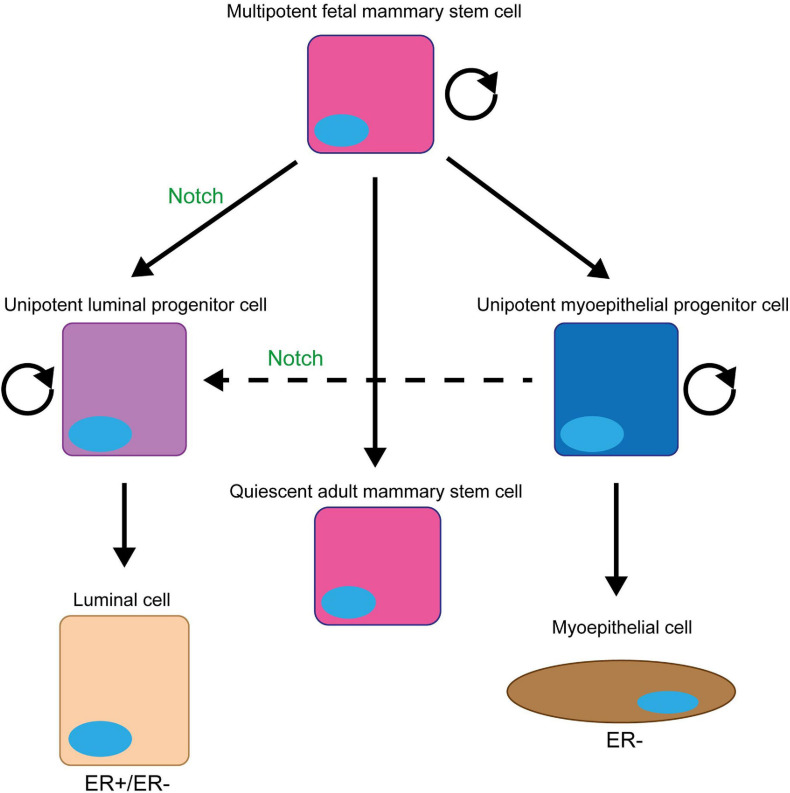
Notch signalling promotes and maintains the luminal progenitor cell fate in the mammary gland. The initial stem cell found within the mammary gland (fMaSC) is multipotent and can form both luminal and myoepithelial cells. However, by late embryogenesis, the gland contains two unipotent progenitor cells that form and maintain the luminal and myoepithelial cell layers of the ductal structures within the gland during puberty and adult life and a quiescent multipotent adult MaSC that is only reactivated upon injury (Woodward et al., 2005; Watson and Khaled, 2020). Notch signalling promotes the differentiation of the foetal MaSCs into the unipotent luminal progenitor cell and prevents this cell differentiating into mature luminal epithelial cells to maintain the population (Dontu et al., 2004; Buono et al., 2006; Bouras et al., 2008; Raouf et al., 2008; Lafkas et al., 2013; Šale et al., 2013; Rodilla et al., 2015; Zhang Y. et al., 2016; Lilja et al., 2018). Upon ablation of the luminal epithelial cells in the adult mammary gland, Notch signalling can also promote the conversion of unipotent myoepithelial progenitor cells into unipotent luminal progenitors to repopulate the luminal lineage (Centonze et al., 2020).

A small subpopulation of mammary epithelial cells can re-populate a full functional mammary gland in a cleared mammary fat pad (Kordon and Smith, 1998; Figure 2). These mammary gland-reconstituting cells contain multi/bipotent mammary stem cells (MaSCs) and unipotent mammary epithelial progenitors (Slepicka et al., 2020). MaSCs are primarily active during the embryonic stage as foetal mammary stem cells (fMaSCs). Most post-natal mammary gland development originates from unipotent lineage-committed progenitors (luminal and myoepithelial progenitor cells) located in the basal epithelium (Inman et al., 2015). Cells in the basal layer generally do not express ER(α), however, luminal cells are a mixed population of both ER+ (oestrogen sensitive) and ER- (oestrogen non-responsive) cells (Clarke et al., 1997; Russo et al., 1999; Watson and Khaled, 2020).

Notch signalling is critical in MaSC and mammary progenitor cell function. Early studies focused on Notch4 signalling suggested that it is upregulated in MaSCs and important for their self-renewal (Dontu et al., 2004; Chakrabarti et al., 2018). However, these studies are not supported by other reports in the literature. Firstly, the original description of the Notch4 knockout mouse failed to identify a mammary gland phenotype (Krebs et al., 2000), suggesting that there isn’t a significant role for Notch4 in MaSCs *in vivo*. Secondly, more recent work looking at the function of the Notch pathway inhibitor Numb in the mammary gland demonstrated that it segregates asymmetrically during MaSC division to the daughter cell with more stem-like characteristics (Santoro et al., 2016), arguing that Notch signalling is blocked in the MaSC. Interestingly, Notch signalling does play an indirect role in maintaining MaSCs within the growing mammary ducts. DLL1 expressed in MaSCs found within the cap cell layer of terminal end buds, the outer layer of cells at growing the tip of mammary duct, activates Notch signalling in adjacent macrophages. This induces the expression of several Wnt proteins which signal back to the MaSCs within the terminal end bud to maintain the stem cell fate (Dontu et al., 2004; Chakrabarti et al., 2018).

On the other hand, there is abundant evidence that Notch signalling plays a significant role in driving MaSCs toward the unipotent luminal progenitor fate. Notch1–3 are more highly expressed in luminal cells, whilst the Notch pathway inhibitors Numb and Numb-like are found in myoepithelial cells (Bouras et al., 2008; Raouf et al., 2008; Raafat et al., 2011; Zhang Y. et al., 2016). Functional studies using knockout and transgenic mouse models and primary human cells have confirmed that Notch signalling controls the luminal vs. myoepithelial lineage balance (Smith et al., 1995; Dontu et al., 2004; Kiaris et al., 2004; Buono et al., 2006; Hu et al., 2006; Bouras et al., 2008; Raouf et al., 2008; Yalcin-Ozuysal et al., 2010; Santoro et al., 2016; Zhang Y. et al., 2016; Onoyama et al., 2020). In the absence of Notch signalling, there is an accumulation of myoepithelial cells, whilst increased Notch signalling leads to an expansion of the of the luminal lineage (Smith et al., 1995; Kiaris et al., 2004; Buono et al., 2006; Hu et al., 2006; Bouras et al., 2008; Yalcin-Ozuysal et al., 2010; Zhang Y. et al., 2016; Onoyama et al., 2020). Lineage tracing studies have found that Notch signalling drives MaSCs into the unipotent luminal progenitor fate by late embryogenesis (Lafkas et al., 2013; Šale et al., 2013; Rodilla et al., 2015; Lilja et al., 2018). However, recent experiments have shown that following ablation of luminal cells, Notch signalling is reactivated in unipotent myoepithelial progenitors to drive the regeneration of luminal cells (Centonze et al., 2020). Interestingly, the functional studies have also shown that Notch signalling not only drives MaSCs toward the luminal progenitor cell fate but also maintains cells in this fate preventing their terminal differentiation (Dontu et al., 2004; Buono et al., 2006; Bouras et al., 2008; Raouf et al., 2008; Zhang Y. et al., 2016). Maintaining this proliferative cell fate could explain why tumour development is seen in transgenic and knockout mouse models where Notch signalling is activated in the mammary gland (Smith et al., 1995; Kiaris et al., 2004; Hu et al., 2006).

Although Notch1, 2, and 3 are all expressed in luminal epithelial cells, Notch3 is the most highly expressed (Raafat et al., 2011). It also appears to be the most important for the decision to adopt the luminal progenitor fate, as the only reports of a phenotype seen when ablating signalling through an individual Notch receptor come from papers reporting the *Notch3* knockout in mice (Xiong et al., 2020) and *Notch3* knockdown in primary human breast epithelial cells (Dontu et al., 2004; Buono et al., 2006; Bouras et al., 2008; Raouf et al., 2008; Zhang Y. et al., 2016). In contrast, expressing an activated form of Notch 1, 3, or 4 seems to be sufficient to drive tumour formation (Smith et al., 1995; Kiaris et al., 2004; Hu et al., 2006; Bouras et al., 2008; Zhang Y. et al., 2016; Onoyama et al., 2020).

## Notch in Breast Cancer

Notch signalling is aberrantly activated in breast cancer, with increased NICD accumulation and target gene expression detected in a range of breast cancer cell lines and primary samples (Weijzen et al., 2002; Stylianou et al., 2006; Mittal et al., 2009). Overexpression of Notch receptors and ligands have been reported in breast tumours, and is correlated with poorer patient prognosis (Reedijk et al., 2005). Aberrant Notch signalling has also been extensively linked to the triple negative breast cancer (TNBC) subtype; Notch receptor overexpression is correlated with the aggressive, metastatic and therapy resistance phenotype characteristic of TNBC (Zhong et al., 2016; Giuli et al., 2019). Notch4 is particularly associated with TNBC. One study found that Notch4 was expressed in 55.6% of TNBC samples compared to 25.5% of ER+ samples (Wang J.W. et al., 2018a).

Data suggests that deregulation of Notch signalling is an early event in breast cancer tumorigenesis, with accumulation of NICD and increased Hey1 expression detected in a broad range of subtypes, including ductal carcinoma *in situ* and epithelial hyperplasia (Stylianou et al., 2006; Mittal et al., 2009; Zardawi et al., 2010). This implies that aberrant Notch signalling plays a causative role in breast tumour initiation. In contrast to haematological malignancies, aberrant activation of Notch signalling in the breast is primarily induced through means other than Notch receptor or ligand mutation, although some mutations have been identified. Activating mutations within and surrounding the PEST domain of Notch1, 2, and 3; mutations disrupting the NRR and heterodimerisation domains; and focal amplifications have been identified in patient tumours and patient-derived xenograft (PDX) models, notably with enrichment in TNBCs (Wang et al., 2015). These mutations result in increased nuclear accumulation of NICD and upregulated target gene expression. In particular, Notch4 mutation and overexpression is correlated with metastatic and poor prognosis TNBC, implicating Notch4 in BCSC activity and chemoresistance (Giuli et al., 2019). Loss of Numb is a frequent cause of aberrant Notch signalling in breast cancer (Stylianou et al., 2006). Pece et al. (2004) found that Numb protein was completely lost or reduced in ∼50% of all breast cancers analysed, through ubiquitination and proteasomal degradation. Numb levels and tumour grade were inversely correlated, which was corroborated by another study that identified Numb loss as a determinant in aggressive and poor prognosis tumours. Collectively, these studies emphasise the importance of Numb as a tumour suppressor in the breast (Colaluca et al., 2008).

Increased Notch activation is sufficient to induce mammary gland tumour formation *in vivo* (Smith et al., 1995; Kiaris et al., 2004; Hu et al., 2006). Moreover, *in vitro*, overexpression of NICD1/4 or RBPJκ-VP16 (which activates RBPJκ-dependent Notch target gene expression in the absence of upstream stimulation) is sufficient to transform mammary epithelial cells (Imatani and Callahan, 2000; Stylianou et al., 2006). Notch co-operation with other pro-tumorigenic signalling pathways, including other developmental pathways, growth factor signalling, pro-inflammatory cytokines, oncogenic kinase pathways and transcription factors, compounds its role in breast tumour initiation and the cancer cell phenotype (Guo et al., 2011). Notch-Wnt crosstalk in particular has been implicated in breast tumour initiation. For example, Ayyanan et al. (2006) demonstrated that Wnt-induced primary mammary epithelial cell transformation was dependent on upregulated Notch activity via increased expression of DLL ligands. Conversely, inhibition of Notch signalling has consistently been shown to reduce or abolish breast tumour development and/or progression (Lee et al., 2008a; Efferson et al., 2010; Castro et al., 2015; Choy et al., 2017). More detail on the potential of Notch therapeutic targeting in cancer will be given later in this review.

## Cell Proliferation

**FIGURE 3:**
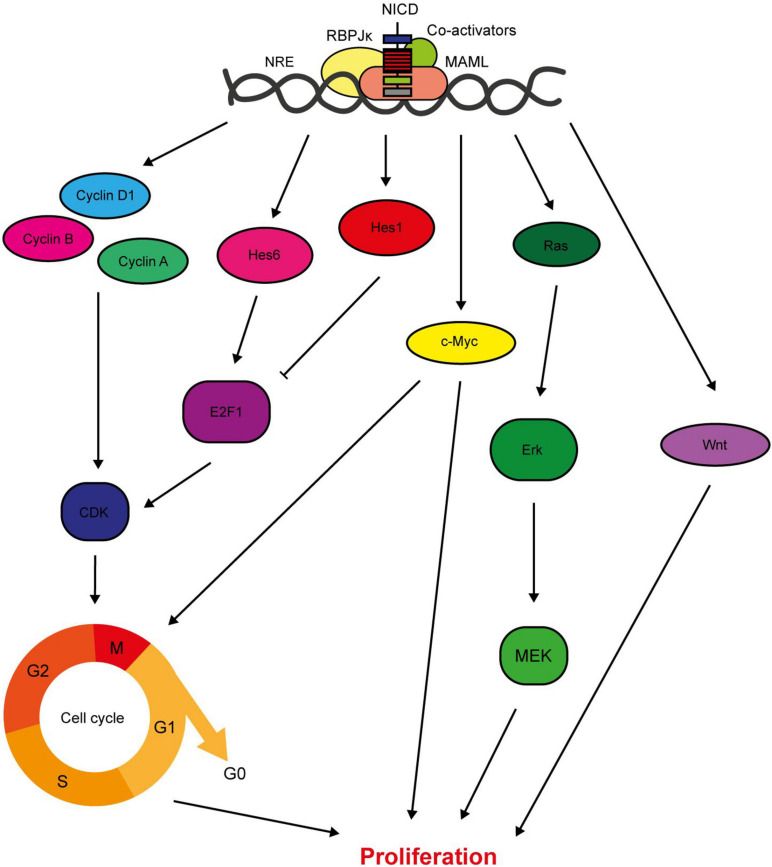
Notch regulates breast cancer cell proliferation. Notch signalling has several direct target genes implicated in cell cycle regulation. These include cyclins A, B and D1, and Hes/Hey family members (Rizzo et al., 2008; Cohen et al., 2010). While most factors downstream of Notch increase the proliferative rate of the cell, Hes1 downregulates E2F1 expression which inhibits cell cycle progression (Hartman et al., 2009; Strom et al., 2009). Notch also activates key oncogenic signalling pathways with pleiotropic effects on cellular function including proliferation, such as c-Myc, Ras and Wnt (Mittal et al., 2009; Aster et al., 2017; Lai et al., 2018).

Signalling from the Notch1, 3, and 4 receptors promotes cell proliferation, both directly through target gene expression and indirectly through activation of downstream signalling pathways (Figure 3). Importantly for the consideration of therapeutic targeting, inhibition of Notch signalling suppresses breast cancer cell proliferation and tumour growth, while ectopic activation of Notch signalling increases proliferation rate (Sun et al., 2005; Yamaguchi et al., 2008; Nagamatsu et al., 2014; Castro et al., 2015; Pei and Wang, 2015; Zhang Q. et al., 2016; Choy et al., 2017; Kong et al., 2018; Rui et al., 2018; Tu et al., 2019). The Hes/Hey canonical Notch target genes are both pro- and anti-proliferative, with Hes1 inhibiting cell cycle progression by suppressing E2F1 expression and Hes6 upregulating E2F1 expression to promote cell cycle progression (Hartman et al., 2009; Strom et al., 2009). In fact, several Notch target genes are cell cycle regulators, meaning that aberrant Notch signalling significantly deregulates cell cycle progression. A few cyclins are upregulated by Notch signalling, and Cyclin D1 is a direct target of JAG1-Notch1/3 signalling in triple negative breast cancer cells (Reedijk, 2012). Inhibition of JAG1 expression in MDA-MB-231 cells is sufficient to reduce cell cycle progression, while JAG1 and cyclin D1 expression are positively correlated in basal breast cancers (Cohen et al., 2010). The proto-oncogene c-Myc is an important direct RBPJκ-dependent Notch target gene. Ablation of c-Myc in MMTV/NICD1 mice can prevent tumour formation (Klinakis et al., 2006; Aster et al., 2017). Crosstalk with signalling molecules such as Ras and Wnt also mediate the role of Notch in breast cancer cell proliferation, with studies detecting concomitant suppression of these pathways in response to Notch inhibition (Mittal et al., 2014; Lai et al., 2018).

## Cell Survival

**FIGURE 4:**
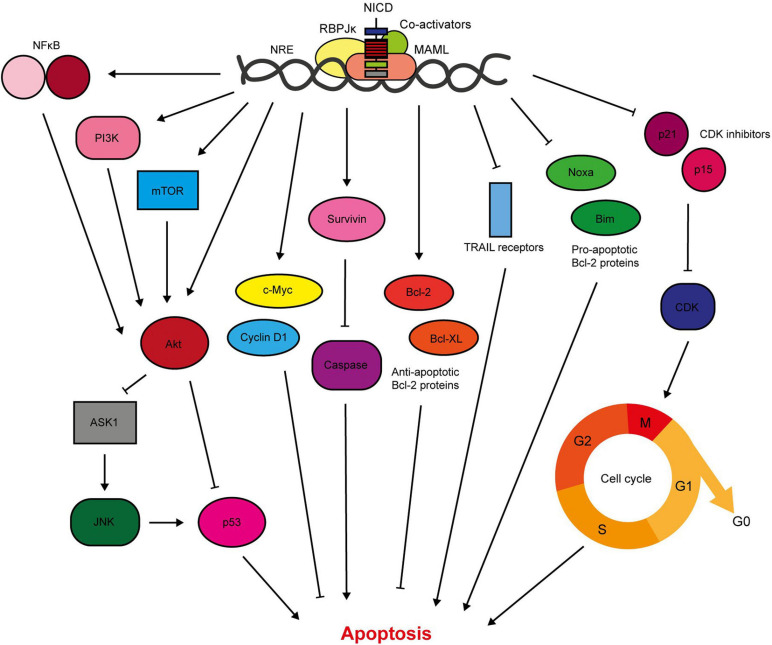
Notch signalling inhibits breast cancer cell apoptosis. Notch activates pro-survival Akt signalling through NFκB, PI3K, and mTOR signalling (Mungamuri et al., 2006; Osipo et al., 2008a; Efferson et al., 2010; Zang et al., 2010; Zhu et al., 2013; Li L. et al., 2014b; Hossain et al., 2018). Our lab have shown that Notch also stimulates Akt through a secreted factor, which triggers stabilisation of p53 through ASK1/JNK signalling (Meurette et al., 2009). The Notch target gene c-Myc is anti-apoptotic, and there is significant evidence demonstrating upregulation of survivin in response to Notch activation (Klinakis et al., 2006; Lee et al., 2008a, b). Survivin prevents apoptosis through indirect and direct caspase inhibition (Garg et al., 2016). Of the Bcl-2 family members, Notch upregulates the anti-apoptotic members including Bcl-2 and Bcl-X_L_, while downregulating pro-apoptotic members such as Bim and Noxa (Portanova et al., 2013; Sales-Dias et al., 2019). Active Notch signalling reduces the sensitivity of TNBC cells to TRAIL-induced apoptosis (Portanova et al., 2013). Notch regulation of cell cycle regulators, including cyclin D1, p21 and p15, also contributes to apoptosis resistance (Cohen et al., 2010; Sales-Dias et al., 2019).

Notch1/3/4 signalling is anti-apoptotic in the breast, and hence promotes breast cancer cell survival (Figure 4). Previous work in our lab has shown that activation of Notch signalling in non-transformed breast epithelial cells inhibits drug-induced apoptosis. Conversely, inhibition of Notch in breast cancer cells is sufficient to re-sensitise the cells to apoptosis. This was determined to be through Notch-induced activation of Akt, via an unknown autocrine signalling factor, and a downstream apoptosis signal regulating kinase 1 (ASK1)/c-Jun N-terminal kinase (JNK)/p53 signalling axis (Stylianou et al., 2006; Meurette et al., 2009). This mechanism was independent of PTEN, an important negative regulator of Akt activation which is downregulated by Notch in other cancer types (Palomero et al., 2007). This work intertwines Notch, Akt and p53 in anti-apoptotic signalling. This supports previous findings by researchers such as Mungamuri et al. (2006) who showed that treatment of Notch-activated MCF-7 cells with a PI3K or mTOR inhibitor sensitised the cells to cytotoxic drug-induced apoptosis, which was accompanied by activation of a p53-specific reporter. Mechanistically, it was determined that Notch1-induced pro-survival signalling was mediated by mTOR-dependent PI3K/Akt inhibition of p53 (Mungamuri et al., 2006).

Furthermore, Notch can activate pro-survival Akt signalling through NFκB in breast cancer cells. Cytoplasmic Notch and phosphorylated Akt (pAkt) correlate with nuclear NFκB in TNBC tumour samples, while mechanistic work in triple negative cell lines demonstrated JAG1-Notch1 (but RBPJκ-independent) stimulation of Akt via mTOR and IκB kinase (IKK) α. Combination treatment of a GSI with either an Akt inhibitor or an IKK inhibitor significantly inhibited TNBC PDX-derived mammosphere growth (Zhu et al., 2013; Hossain et al., 2018). This corroborates data from the Liu lab which showed that Notch1-induced proliferation and reduction in apoptosis was accompanied by NFκB activation and target gene expression in triple negative MDA-MB-231 cells (Li L. et al., 2014b).

Additionally, Notch upregulates anti-apoptotic proteins such as survivin and Bcl2 (Lee et al., 2008b; Sales-Dias et al., 2019). Lee et al. (2008a) showed that GSI treatment reduced survivin expression in triple negative breast cancer cell lines (but not ER+ cell lines), which was sufficient to induce apoptosis, prevent colony formation in soft agar and inhibit xenograft tumour growth and lung metastasis in mice. Portanova et al. (2013) also demonstrated sensitisation of breast cancer cell lines to tumour necrosis-factor related apoptosis-inducing ligand (TRAIL)-induced apoptosis by GSI treatment. Interestingly this effect was found to be more potent in the triple negative MDA-MB-231 cell line compared to ER+ MCF-7 cells. TNBC is the most common breast cancer subtype that develops in BRCA1 mutation carriers (Mavaddat et al., 2012). In a high throughput sequencing screen of BRCA1-deficient murine mammary tumours, Notch1 was identified as an oncogenic driver. Notch1 suppressed DNA damage and mitotic catastrophe (and therefore lethality) caused by BRCA1 deficiency. Notch1 signalling restored the S/G2 and G2/M cell cycle checkpoints, likely through an ATR/CHK1 signalling pathway (Miao et al., 2020).

## EMT, Invasion and Metastasis

Notch signalling is implicated in a broad range of processes required for breast cancer cell metastasis including survival of hypoxia, angiogenesis, EMT, local tissue invasion, survival in the circulation, chemoresistance and colonisation of secondary sites (Zhang et al., 2019).

**FIGURE 5:**
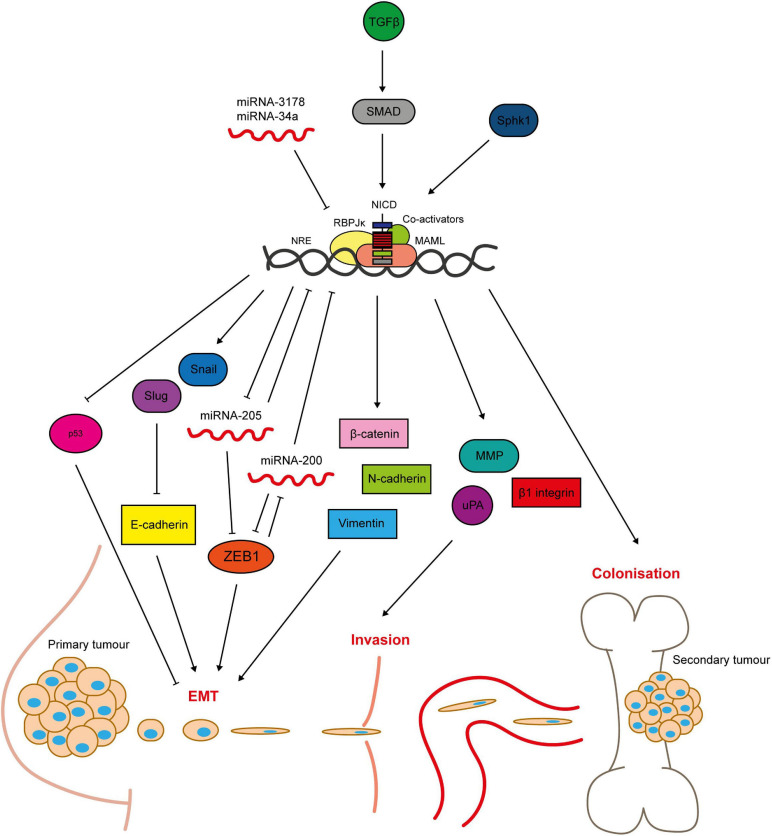
Notch signalling regulates breast cancer cell metastasis. Notch-mediated metastasis is induced by factors such as TGFβ and Sphk1 (Zavadil et al., 2004; Zhang et al., 2010; Sethi et al., 2011; Wang S. et al., 2018b). Notch activates key regulators of EMT including the transcriptional repressors Slug and Snail, that mediate loss of cell-cell contacts through inhibition of E-cadherin expression (Leong et al., 2007; Sahlgren et al., 2008; Zhang et al., 2010; Du et al., 2012; Shao et al., 2015; Kong et al., 2018). The mesenchymal markers ZEB1, β-catenin, N-cadherin and vimentin are upregulated by Notch signalling (Bolós et al., 2013; Lai et al., 2018). ZEB1 is activated through complex bi-directional signalling involving micro-RNAs (Brabletz et al., 2011). Micro-RNAs negatively regulate Notch signalling, and their loss is sufficient to induce EMT in breast epithelial cells (Du et al., 2012; Chao et al., 2014; Kong et al., 2018). Notch is also implicated in tissue invasion, as it upregulates matrix-degrading enzymes including MMP2 and 9 and urokinase-type plasminogen activator (uPA), as well as β1-integrin (Shimizu et al., 2011; Lai et al., 2018). Anti-apoptotic Notch signalling (see Figure 4) enables the cells to survive in the blood stream and travel to secondary sites. Notch signalling between the cancer cells and cells in the bone microenvironment facilitates colonisation and growth at the metastatic site (Sethi et al., 2011).

Notch signalling promotes breast epithelial cell EMT (Leong et al., 2007). Inhibition of Notch signalling in TNBC cells reverses the characteristic epithelial to mesenchymal cobblestone to spindle cell morphology and associated marker switch, as well as reducing invasion and migration (Shao et al., 2015; Zhang J. et al., 2016). Mechanistically, Notch induces EMT through activation of Slug and Snail; transcriptional repressor proteins that downregulate E-cadherin expression (Martin et al., 2005; Leong et al., 2007; Zhang et al., 2010; Shao et al., 2015; Figure 5). JAG1, Notch1 and Slug expression correlate in patient tumour samples. Notch4 inhibition also reduces the number and size of MDA-MB-231 xenograft tumour metastases *in vivo*, which is accompanied by restoration of E-cadherin expression, inactivation of β-catenin and downregulation of Slug (Leong et al., 2007). Interesting recent work suggests that differences between Notch/DLL and Notch/JAG signalling may induce different patterns of EMT within a cancer, with Notch/DLL signalling favouring the induction of EMT in individual cells and Notch/Jag signalling favouring EMT in clusters of cells (Bocci et al., 2019, 2020). The latter may go on to form circulating tumour cell clusters.

Hypoxia-induced breast epithelial cell EMT appears to be dependent on Notch signalling, with one study finding that hypoxia downregulated E-cadherin in MCF10A cells only when Notch signalling was aberrantly activated by immobilised JAG1 (Sahlgren et al., 2008). Another study found that Snail expression and E-cadherin downregulation induced by hypoxic treatment of breast cancer cells was abrogated by GSI treatment or dominant negative MAML expression. Hypoxic breast cancer cells had increased invasive and migratory capability in Boyden chamber and scratch wound assay respectively, which was reversed by GSI treatment (Chen et al., 2010). Notch1 has been implicated specifically in triple negative breast cancer EMT. For example, Notch1 is a downstream target of miRNA-3178, an inhibitory miRNA downregulated in TNBC (but not non-TNBC subtypes), that plays a role in EMT through regulation of Snail (Kong et al., 2018). Micro-RNAs are a recurring theme in Notch-mediated EMT, with miRNAs acting both upstream and downstream of the pathway (Du et al., 2012; Chao et al., 2014; Figure 5). Notch is also implicated in bi-directional crosstalk with the mesenchymal marker zinc finger E-box binding homeobox 1 (ZEB1). Knockdown of ZEB1 in breast cancer cells inhibits Notch activity, including downregulation of JAG1, MAML2/3 and HEY1 expression, via de-repression of miRNA-200 expression. In primary TNBC samples high ZEB1 expression was correlated with upregulated JAG1 and Notch activity in invasive tumour regions (Brabletz et al., 2011).

The role of Notch signalling in EMT corresponds to its promotion of invasive and metastatic phenotypes. Activation of Notch signalling in non-invasive breast cancer cells promotes cell invasion and migration, while inhibition of Notch in invasive cells reduces their invasive and migratory capacity (Bolós et al., 2013; Castro et al., 2015; Lai et al., 2018; Leontovich et al., 2018). Moreover, Notch signalling is correlated with metastasis *in vivo* (Kontomanolis et al., 2014). In a single cell gene expression analysis, NOTCH4, NOTCH3 and JAG1 were upregulated in metastatic breast cancer cells compared to primary tumour cells isolated from TNBC patient-derived xenograft (PDX) models (Lawson et al., 2015). JAG1-induced Notch signalling is also important in breast cancer cell colonisation of the bone metastatic niche (Zhang et al., 2010). High JAG1 expression is correlated with bone-tropic metastatic breast cancer cell lines and samples from patient bone metastasised tumours. It was shown that JAG1 is upregulated in the cancer cells by SMAD-dependent TGFβ signalling (Figure 5), and activates Notch signalling in osteoblasts within the bone microenvironment. Importantly, pharmacological inhibition of Notch signalling was sufficient to reduce breast cancer bone metastasis and osteolysis *in vivo*, implying that targeting Notch signalling may be a suitable therapeutic approach for inhibiting breast cancer metastasis (Sethi et al., 2011).

## A Role in Breast Cancer Therapy Resistance

Notch signalling is induced by breast cancer chemotherapy, and is upregulated in therapy-resistant tumour cells (Bhola et al., 2016; Xiao et al., 2019). Activation of Notch signalling is sufficient to induce chemotherapy resistance, while inhibition of Notch signalling re-sensitises resistant cells to conventional therapy (Li X.J. et al., 2012a; García-Heredia et al., 2016; Xiao et al., 2019). Combining Notch inhibitors with conventional chemotherapies has an additive effect, increasing treatment efficacy both *in vitro* and *in vivo* (Qiu et al., 2013; Rustighi et al., 2014; Li et al., 2015; Zhou et al., 2017). Furthermore, the failure of inhibitors of key pro-oncogenic signalling pathways in clinical trials has been partially attributed to Notch signalling. For example, investigation of TNBC PI3K/mTOR inhibitor resistance found that PI3K/mTOR or TORC1/2 treatment enriched for BCSCs with upregulated Notch1 expression. GSI Notch blockade prevented this BCSC enrichment (Bhola et al., 2016). In addition, Diluvio et al. (2018) demonstrated that GSI treatment sensitised EGFR tyrosine kinase inhibitor (TKI) resistant TNBC cells to gefitinib.

Notch signalling is also implicated in breast cancer cell resistance to radiotherapy. Radiation induces BCSCs and Notch activity *in vivo* (Lagadec et al., 2013; Kim et al., 2016), which confers radioresistance in TNBC (Lee et al., 2019). GSI treatment prevents radiation-induced BCSC enrichment (Lagadec et al., 2013). Mechanistically, Notch has been found to mediate triple negative/basal-like breast cancer radioresistance through BCSC enrichment downstream of tribbles homolog 3 (TRIB3), and in parallel with STAT1 (Boelens et al., 2014; Lee et al., 2019). It also mediates radiation-induced EMT as part of an IL-6/JAK/STAT3 signalling axis (Kim et al., 2016).

There is significant evidence to suggest that Notch signalling plays a role in ER+ breast cancer endocrine therapy resistance (Acar et al., 2016). Notch signalling is upregulated in endocrine therapy resistant ER+ breast cancer cell lines (Magnani et al., 2013). In particular, Notch4 activity has been found to be increased in resistant ER+ cell lines and tamoxifen and fulvestrant-treated PDX models. Activation of JAG1/Notch4 signalling was sufficient to induce endocrine therapy resistance in MCF-7 cells, and tamoxifen resistance could be predicted for in ER+ breast cancer patients using a Notch4/HES/Hey gene signature. This Notch4-induced resistance was accompanied by an enrichment for BCSCs. GSI treatment inhibited endocrine therapy-induced BCSC activity and re-implantation tumour formation in breast cancer PDX models and cell lines (Simões et al., 2015). Furthermore, evidence suggests that Notch signalling functions in a paracrine signalling mechanism between bulk ER+ tumour cells and ER- BCSCs (Harrison et al., 2013).

Finally, Notch signalling has been connected to trastuzumab and lapatinib resistance in HER2+ breast cancer. Notch signalling is upregulated following trastuzumab or lapatinib treatment and HER2 positive cells have lower Notch transcriptional activity than HER2 negative cells (Osipo et al., 2008b; Abravanel et al., 2015). This is controlled by several mechanisms, including through HES1 and NRARP, but also through protein kinase C (PKC) α, which acts downstream of HER2 to restrict the availability of JAG1 for receptor binding (Abravanel et al., 2015). Interestingly, PKCα/Notch4 crosstalk has also been identified in endocrine therapy resistant ER+ breast cancers (Yun et al., 2013). Regardless, trastuzumab inhibits HER2, so trastuzumab treatment releases the block on Notch activation, enabling the cells to survive. Importantly, combining trastuzumab with a GSI potentiates the HER2-targeting treatment, and restores sensitivity to resistant cells (Osipo et al., 2008b; Pandya et al., 2016). Trastuzumab/GSI combination treatment also prevented breast tumour recurrence post-treatment insensitive orthotopic breast tumour xenografts (Pandya et al., 2011).

## Breast Cancer Stem Cells

**FIGURE 6:**
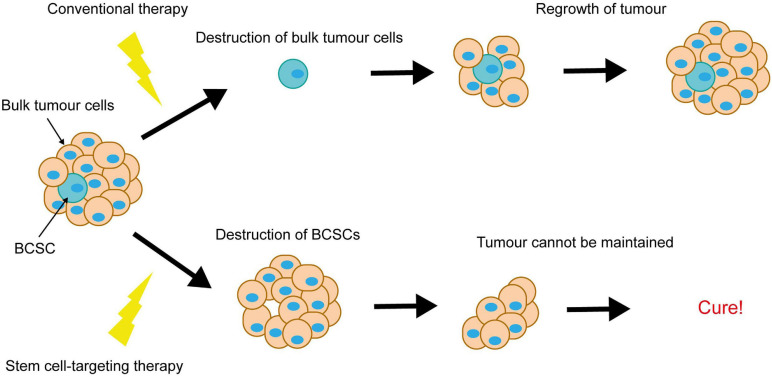
Elimination of breast cancer stem cells is key to achieving complete tumour regression. Conventional therapies destroy the bulk tumour cells, causing tumour regression, however resistant BCSCs survive and re-populate the tumour. Elimination of the BCSCs (even without immediate destruction of the bulk tumour cells) could induce complete tumour regression, as the tumour cells die off and are not replaced.

Notch-conferred therapy resistance is often accompanied by enrichment for breast cancer stem cells (BCSCs). BCSCs are defined as a subpopulation of cancer cells within the breast tumour, capable of both self-renewal and differentiation. They are purportedly responsible for tumour initiation, intratumoral heterogeneity and disease recurrence, and are more resistant to therapy than the rest of the tumour cell population. This puts them in particular focus for the development of novel breast cancer therapies, as ablation of BCSCs would result in tumour regression and eliminate risk of recurrence (Figure 6).

Notch signalling is implicated in BCSC self-renewal. Notch1 expression is positively correlated with ALDH positivity in breast tumour samples, and downregulation of Notch signalling in ALDH+ cells inhibits growth and induces apoptosis (Suman et al., 2013; Zhong et al., 2016). Breast cancer cells with high Notch activity are also more stem cell-enriched, and activation of Notch with DSL peptide increases mammosphere self-renewal in patient-derived samples (Dontu et al., 2004; D’Angelo et al., 2015; Mamaeva et al., 2016).

In line with its connection to endocrine therapy resistance, Notch4 appears to be the most important of the Notch receptors in breast cancer stem cells (Harrison et al., 2010). Notch4 expression is detected in secondary mammospheres, the basal layer in mammary gland tissue samples and is restricted to the terminal end bud (TEB) regions of mammary ducts (Dontu et al., 2004; Harrison et al., 2010). Notch4 blocking antibody treatment reduces primary mammosphere forming efficiency (MFE) and abolishes secondary mammosphere formation (Dontu et al., 2004; Farnie et al., 2007). Notch3 is also involved, with inhibition or ablation of Notch3 sufficient to reduce breast cancer cell mammosphere formation and self-renewal, and BCSC marker expression (Sansone et al., 2007a, b). Mechanistically, Notch signalling may enhance the expansion of BCSCs and/or progenitors through downstream cyclin D1 activity (Ling et al., 2010; Ling and Jolicoeur, 2013). Numerous factors have been identified upstream of Notch-induced BCSC activity including JNK, Ras, Pin1, HIF, cyclooxygenase 2 (COX2), syndecan-1 and BMP-4 (Mittal et al., 2014; Rustighi et al., 2014; Ibrahim et al., 2017; Xie et al., 2017; Yan et al., 2018; Choi et al., 2019). Notch/ZEB1 signalling has recently been implicated in the critical interaction between BCSCs and the tumour microenvironment. JAG1 expressed on endothelial cells in the tumour microenvironment activates Notch1 in adjacent BCSCs, resulting in ZEB1 induction and increased stemness. A positive feedback loop is formed as ZEB1 upregulates endothelial JAG1 via VEGFA (Jiang et al., 2020).

Evidence collected in these studies suggest that targeting Notch signalling as a part of breast cancer therapy may enable us to home in on BCSCs within the tumour cell population. In an exciting study, Mamaeva et al. (2016) developed mesoporous silica nanoparticles (MSNs, functionalised with glucose moieties) designed to specifically target breast cancer cells and BCSCs with γ-secretase inhibitors. They found that these DAPT loaded MSNs reduced the BCSC pool both *in vitro* and *in vivo* (Mamaeva et al., 2016). A note of caution however, lies in the heterogeneity of BCSCs and the implication on treatment efficacy. For example, one study identified two tumour initiating cell subsets within the BCSC population and demonstrated active Notch1 signalling in the more proliferative, invasive and metastatic subpopulation (CD44^+^/CD24^low^) but not the other (CD44^+^/CD24^–^). Concordantly, GSI treatment reduced mammosphere formation and tumour growth from CD44^+^/CD24^low^ cells but not CD44^+^/CD24^–^ cells (Azzam et al., 2013). These data warn that BCSC heterogeneity may limit the efficacy of GSI’s in breast cancer therapy. Despite this concern, studies have shown that Notch inhibitors can still successfully reduce the overall CD44^high^/CD24^low/–^ subpopulation, and that this has phenotypic effect in mammosphere and re-implantation assays, particularly in combination with other agents (Qiu et al., 2013; Mittal et al., 2014; D’Angelo et al., 2015). Importantly, GSI treatment and Notch antibody blockade have been used to inhibit breast cancer cell secondary re-implantation tumour development, alone or in combination with docetaxel (Qiu et al., 2013; D’Angelo et al., 2015).

## Crosstalk With ER and HER2 Signalling

A significant factor when considering the therapeutic value of targeting Notch signalling in the different subtypes of breast cancer, is the pathway’s crosstalk with ER and HER2 signalling. There is a clear correlation between aberrant Notch signalling and the triple negative phenotype, and multiple studies have identified roles for Notch signalling in TNBC that are not recapitulated in ER+ or HER2+ breast cancer (Lee et al., 2008a; Yamaguchi et al., 2008; Giuli et al., 2019). Notch/ER/HER2 crosstalk is also key in Notch-mediated resistance to endocrine and HER2-targeting therapy.

The Miele group have shown that Notch transcriptional activity is highest in ER- breast cancer cells, where inhibition of Notch signalling is effective in inducing cancer cell death *in vitro*. In contrast, in ER+ breast cancer cells, oestrogen inhibits Notch activity. This means that Notch activity is induced by endocrine therapy in the ER+ subtype, contributing to therapy resistance. Combining tamoxifen with a GSI resulted in significantly enhanced ER+ xenograft tumour regression compared to monotherapy, suggesting that combining Notch inhibitors with endocrine therapy may be a promising therapeutic strategy for ER+ breast cancer (Rizzo et al., 2008). Supporting this, mutation of ER in breast cancer stem cells induces Notch4 activity (Gelsomino et al., 2018). Similarly, HER2 suppresses Notch signalling in HER2+ breast cancer cells (Osipo et al., 2008b; Ju et al., 2013). HER2 regulates the activity of γ-secretase via ERK, and the nuclear translocation of NICD via Akt, in independent mechanisms (Ju et al., 2013).

**FIGURE 7:**
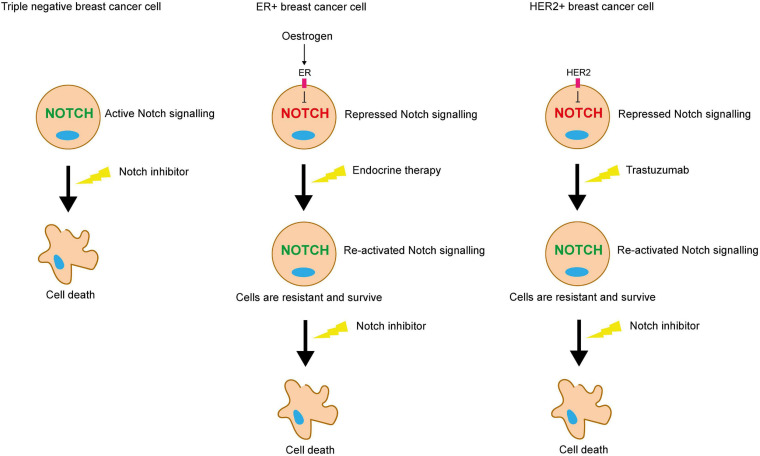
Notch inhibition may be a viable strategy for targeting therapy resistant breast cancer cells. ER and HER2 signalling inhibits Notch in ER+ and HER2+ breast cancer cells respectively. Endocrine or trastuzumab treatment inhibit these pathways, releasing the blockade on Notch signalling. Pro-survival Notch activity enables the cells to survive the targeted treatments. Notch inhibitors could be used in combination to sensitise these resistant cells to the targeted treatment. Triple negative breast cancer cells lack the ER and HER2 receptors, meaning that they are unaffected by endocrine therapy or trastuzumab, but are sensitive to Notch inhibitors.

Collectively, these studies suggest that in the absence of the growth-promoting pathways induced by ER and HER2, whether it be in the context of TNBC or cancers treated with anti-ER or HER2 therapies, Notch acts as a compensatory growth-promoting stimulus, enabling the cells to survive in the absence of these pathways (Figure 7).

Conversely, several studies contradict these findings and provide evidence that ER signalling promotes Notch signalling (Calaf and Roy, 2008; Kumar et al., 2019). Imperfect oestrogen response elements (EREs) have been detected in the Notch1 and JAG1 promoters, which translated to oestrogen-induced expression and increased signalling activity (Soares et al., 2004). Moreover, adding further complexity and implying the presence of regulatory feedback loops, Notch signalling can transactivate lower levels of the ER signalling pathway in the absence of oestrogen (Hao et al., 2010). RBPJκ binding sites have also been identified in the ERα promoter, suggesting that Notch can upregulate ERα expression (Dou et al., 2017). Likewise, Notch1 induces HER2 transcription in a RBPJκ-dependent mechanism (Chen et al., 1997).

## Targeting Notch in Breast Cancer Therapy

Due to its multiple roles in breast tumorigenesis and therapy resistance, the Notch signalling pathway is an attractive therapeutic target. Many studies have shown that pharmacological Notch inhibition is effective both *in vitro* and in *in vivo* mouse models at inhibiting tumour growth, inducing tumour regression, preventing metastasis, targeting BCSCs and sensitising breast cancer cells to conventional therapies.

**FIGURE 8:**
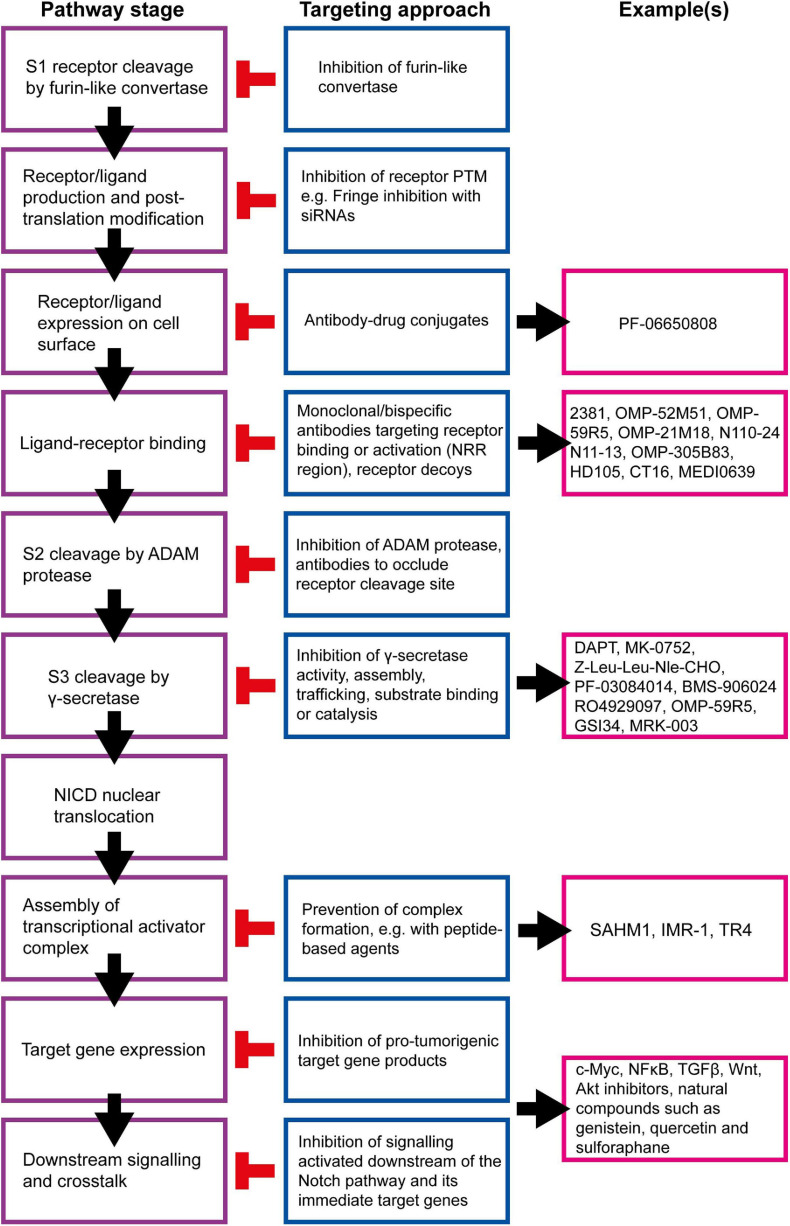
Summary of targetable points of the Notch pathway. The Notch signalling pathway can be inhibited at almost all stages, and a number of strategies are being developed to target these steps for therapeutic purposes (Andersson and Lendahl, 2014). γ-secretase inhibitors (GSIs) are the most well-established Notch inhibitors. Most competitively inhibit presenilin in the γ-secretase complex, and are hence pan Notch inhibitors that prevent all signalling events downstream of the Notch receptor regardless of receptor isoform or activating ligand (Krishna et al., 2019). The γ-secretase complex can also be targeted with monoclonal antibodies raised against presenilin or nicastrin (Hayashi et al., 2012; Takagi-Niidome et al., 2013; Zhang et al., 2014). Other pan Notch inhibitors include those that target the NICD/RBPJκ/MAML transcriptional activator complex. SAHM1 is a synthetic hydrocarbon-stapled α-helical peptide designed to mimic a portion of the N terminus of MAML. It competitively binds NICD/RBPJκ, preventing MAML binding (Moellering et al., 2009). Ligand-receptor binding is a popular target for current Notch inhibitor development. This can be achieved through receptor decoys, monoclonal antibodies, bispecific antibodies and antibody-drug conjugates (Noguera-Troise et al., 2006; Li L. et al., 2008; Hoey et al., 2009; Wu et al., 2010; Sharma et al., 2012; Li D. et al., 2014; Kangsamaksin et al., 2015; ClinicalTrials.gov., 2016, 2019a; Hidalgo et al., 2016; Lee et al., 2016; Cubillo Gracian et al., 2017; Hu et al., 2017; Lamy et al., 2017; Zheng et al., 2017; Hughes et al., 2018; Giuli et al., 2019; Jimeno et al., 2019; Smith et al., 2019; Rosen et al., 2020). Various natural compounds (and their derivatives) have been found to inhibit Notch signalling (Kawahara et al., 2009; Kallifatidis et al., 2011; Li Y. et al., 2012b; Pan et al., 2012; Xia et al., 2012; Nwaeburu et al., 2016; Sha et al., 2016; Mori et al., 2017; Castro et al., 2019; Li et al., 2020). These hold potential to be adapted and appropriated into cancer therapy.

Many strategies for targeting the Notch signalling pathway have been developed and examples of currently available inhibitors are summarised in Figure 8. However, there are several issues that must be considered before these inhibitors are used clinically, beyond the obvious need to select patients with Notch-responsive tumours. These issues may explain why many clinical trials involving the use of Notch inhibitors have been put on hold or terminated due to toxicity or failure to reach trial endpoints, despite showing promise in pre-clinical studies (Table 1; Mollen et al., 2018).

**TABLE 1:** Notch inhibitor clinical trials featuring breast cancer patients.

Notch inhibitor	Status	Dose	Mono/combination therapy	Breast cancer (sub)type	Phase	Number of participants	Results	Identifier/references
MK-0752 (GSI)	Completed	Various	Monotherapy	Advanced breast cancer	I	103	Weekly dosing well tolerated (schedule dependent toxicity), significant inhibition of Notch signalling	NCT00106145 (Krop et al., 2012)
	Completed	Escalating doses, daily on days 1–3	Combination with docetaxel (chemotherapeutic) and pegfilgrastim (GCSF analog)	Advanced/metastatic breast cancer	I/II	30	Manageable toxicity, preliminary evidence of efficacy	NCT00645333 (Schott et al., 2013)
	Completed	350 mg daily, 3 days on 4 days off, etc. for 10 days total	Combination with tamoxifen (selective oestrogen receptor modulator) or letrozole (aromatase inhibitor)	Early stage breast cancer (pre-surgery)	IIII	22	75% of participants experienced non-serious adverse events	NCT00756717
PF-03084014 (GSI)	Terminated (change in drug development strategy)	80–150 mg twice daily	Combination with docetaxel	Advanced/metastatic breast cancer	I	30	N/A	NCT01876251
	Terminated (change in drug development strategy)	150 mg twice daily	Monotherapy	Advanced TNBC	II	19	N/A	NCT02299635
	Withdrawn (drug discontinued)	150 mg daily on days 1 and 9 or twice daily on days 2–8	Monotherapy	Non-metastatic TNBC with chemoresistant residual disease	II	0	N/A	NCT02338531
AL101 [formerly BMS-906024 (GSI)]	Completed	Various	Monotherapy	Refractory/relapsed breast cancer	I	94	Generally well tolerated, sustained Notch inhibition, demonstrated clinical activity	NCT01292655 (El-Khoueiry et al., 2018)
	Completed	4 or 6 mg weekly every 3 weeks	Combination with chemotherapy regimes involving paclitaxel, 5-fluorouracil, carboplatin, leucovorin and irinotecan	Advanced/metastatic (breast cancer preferred)	I	141	Unavailable	NCT01653470
	Recruiting	6 mg weekly	Monotherapy	Notch-activated recurrent/metastatic TNBC	II	67	N/A	NCT04461600 (Traina et al., 2021)
LY3039478 (GSI)	Completed	Up to maximum tolerated dose, various schedules	Combination with taladegib (Hedgehog inhibitor) or abemaciclib (CDK inhibitor)	Advanced/metastatic breast cancer	I	94	Poorly tolerated, disappointing clinical activity	NCT02784795 (Azaro et al., 2021b)
	Completed	Various (escalating)	Monotherapy or in combination with prednisone (corticosteroid)	Advanced/metastatic breast cancer	I	237	Well tolerated at doses where Notch inhibition was detected, clinical activity observed	NCT01695005 (Massard et al., 2018; Azaro et al., 2021a)
RO4929097 (GSI)	Terminated	Escalating dose on day 1 or days −2, −1, and 1 of course 1 and days 1–3 and 8–10 of course 2 and all subsequent courses	Combination with vismodegib (Hedgehog inhibitor)	Metastatic breast cancer	I	13	N/A	NCT01071564
	Terminated	Daily on days 1–3, 8–10, and 15–17, every 21 days for 6 courses	Combination with neo-adjuvant paclitaxel and carboplatin (chemotherapeutics)	Stage II/III TNBC	I	14	N/A	NCT01238133
	Terminated (slow accrual, drug discontinued)	Up to maximum tolerated dose	Combination with whole-brain radiotherapy or stereostatic radiosurgery	ER- breast cancer metastasised to the brain	I	5	N/A	NCT01861054
	Terminated	Daily on days 1–3, 8–10, and 15–18, every 21 days for 6 courses	Combination with letrozole	Post-menopausal, stage II/III breast cancer	I	28	N/A	NCT01208441
	Completed	Daily on days 1–3, 8–10, and 15–17, course repeats every 21 days	Combination with capecitabine (chemotherapy)	Refractory breast cancer	I	30	Unavailable	NCT01158274
	Terminated (drug development discontinued)	Daily on days 1–3, 8–10, and 15–17, course repeats every 21 days	Combination with exemestane (aromatase inhibitor)	Advanced/metastatic breast cancer	I	15	N/A	NCT01149356
	Completed	Daily on days 1–3, 8–10, and 15–17 (days 1–3, 8–10, 15–17 22–24, 29–31, and 36–38 of course 1 only), course repeats every 21 days	Combination with cediranib maleate (VEGF inhibitor)	Advanced breast cancer	I	20	Unavailable	NCT01131234
	Terminated (slow accrual, drug discontinued)	Up to maximum tolerated dose	Combination with whole-brain radiation therapy or stereotactic radiosurgery	Breast cancer metastasised to the brain	I	5	N/A	NCT01217411
	Terminated (low enrolment)	Once daily days 1–3, 8–10 and 15–17, every 21 days	Monotherapy	Advanced/metastatic/recurrent TNBC	II	6	N/A	NCT01151449
CB-103 (transcriptional activator complex inhibitor)	Recruiting	Daily	Combination with non-steroidal aromatase inhibitor [(NSAI) anastrozole or letrozole]	Advanced ER+/HER2- breast cancer with prior NSAI benefit	II	80	N/A	NCT04714619
	Recruiting	15 mg daily	Monotherapy	Advanced/metastatic breast cancer	I/II	165	N/A	NCT03422679
OMP-52M51 (anti-Notch1 monoclonal antibody)	Completed	Up to maximum tolerated dose	Monotherapy	Refractory breast cancer with evidence of Notch1 activation	I	48	Well tolerated	NCT01778439 (Ferrarotto et al., 2018)
OMP-59R5 (anti-Notch2/3 cross-reactive antibody)	Completed	Up to maximum tolerated dose (<2.5 mg weekly and 7.5 mg/kg every other and every third week)	Monotherapy	Metastatic breast cancer	I	42	Generally well tolerated up to the maximum tolerated dose, demonstrable Notch inhibition	NCT01277146 (Smith et al., 2019)
PF-06650808 (anti-Notch3 monoclonal antibody)	Terminated (change in sponsor prioritisation)	0.2 mg/kg to maximum tolerated dose	Monotherapy	Advanced breast cancer	I	40	Manageable safety profile, preliminary signs of anti-tumour activity	NCT02129205 (Rosen et al., 2020)

*Information obtained from ClinicalTrials.gov, reference provided where publication available.*

Firstly, Notch signalling is a ubiquitous and essential developmental signalling pathway. It functions in normal tissue homeostasis throughout the body, meaning that systemic inhibition could have potentially harmful effects in healthy organs and mammary gland tissue. For example, long term γ-secretase inhibition has been shown to cause significant histopathologic changes in the gastrointestinal (GI) tract, including intestinal goblet cell metaplasia, apoptosis of small intestinal crypt epithelial cells, villous stunting, epithelial vacuolation and accumulation of intraluminal mucous (Milano et al., 2004). Another study found that long term anti-DLL4 antibody treatment in mice and rats caused highly significant histopathological defects in multiple organs including the liver and thymus. Most detrimentally, was the formation of ulcerating subcutaneous tumours with features of vascular neoplasms. Rarer necrotic lesions were also identified in the heart and lungs (Yan et al., 2010).

Secondly, targetable proteins within the Notch pathway are shared with other pathways important in normal cellular function. For instance, γ-secretase cleaves many other substrates including low density lipoprotein (LDL) receptor-related protein, E-cadherin, ErbB-4 and amyloid-β protein precursor (AβPP) (Haapasalo and Kovacs, 2011).

Thirdly, particularly in the case of cancer therapy, Notch functions as a tumour suppressor in certain contexts. This means that patients treated with systemic pan Notch inhibitors may be in danger of secondary tumour development, an unacceptable risk. Studies have found evidence for tumour suppressive Notch in a number of tissues, but this is most well characterised in the skin (Nicolas et al., 2003). Loss of function Notch1 mutations have been identified in squamous cell carcinoma, where they occur early on in tumorigenesis (Agrawal et al., 2011; Wang et al., 2011; South et al., 2014). The clinical significance of this was demonstrated in a phase III trial of the GSI semagacestat for Alzheimer’s disease. In addition to failing to slow disease progression, semagacestat increased the risk of skin cancer in the treated patient cohort (Extance, 2010). It may also be the case that Notch has contrasting roles within the same tissue, dependent on factors such as receptor isoform, the strength of activation signal and the presence or absence of regulators (Nowell and Radtke, 2017). Within breast cancer, the role of Notch2 has been controversial, with some studies finding that it has a tumour suppressive role, in direct contrast to the Notch1 and 4 isoforms (Parr et al., 2004; O’Neill et al., 2007). Similarly, Notch3 has been found to inhibit breast cancer cell EMT (Zhang X. et al., 2016; Lin et al., 2018; Wen et al., 2018).

There are a number of strategies that have been suggested for overcoming the gastrointestinal side effects associated with pan Notch inhibition. These include minimal dosing and intermittent administration to reduce the length of continuous treatment periods, which has shown some success (Krop et al., 2012). Z-Leu-Leu-Nle-CHO can be used at lower doses than conventional GSIs, as it is also anti-tumorigenic through negative regulation of the proteasome (Han et al., 2009). Moreover, nanoparticles could be utilised to specifically direct GSIs to the tumour, avoiding damage to healthy tissue (Mamaeva et al., 2016).

GSIs could be combined with conventional chemotherapy and targeted treatments. This has the dual benefit of minimising of the dose of each individual drug required (reducing toxicity), and enhancing overall treatment efficacy. Notch inhibition alone is unlikely to be sufficient to induce tumour regression, but shows promise in combination therapy (Meurette et al., 2009; Brennan and Clarke, 2013; Proia et al., 2015). In clinical trials, GSIs have been combined with conventional chemotherapeutics and endocrine therapy, as well as radiotherapy to help improve radiosensitivity and reach metastatic cells in hard to access areas (Table 1; Albain et al., 2010; ClinicalTrials.gov., 2016, 2019a,b; Lamy et al., 2017).

An alternative strategy to minimise the risk of side effects is to target specific Notch receptors or ligands. Monoclonal antibodies have been developed that bind to specific Notch receptor/ligand isoforms to inhibit receptor-ligand interaction, prevent processing by ADAM proteases, or induce inactivation via a conformational change of the receptor structure (Giuli et al., 2019; see Figure 8 for examples). These have shown promising results in pre-clinical studies, but have suffered end point failure in clinical trials (Noguera-Troise et al., 2006; Li L. et al., 2008; Hoey et al., 2009; Wu et al., 2010; Sharma et al., 2012; Li D. et al., 2014; ClinicalTrials.gov., 2016, 2019a; Hidalgo et al., 2016; Cubillo Gracian et al., 2017; Lamy et al., 2017; Zheng et al., 2017; Hughes et al., 2018; Giuli et al., 2019; Smith et al., 2019). Bispecific antibodies and antibody-drug conjugates may be able to improve on the limited success of monoclonal antibodies. Bispecific antibodies can be used to target other oncogenic signalling pathways simultaneously, which has been proven to be more efficacious than administering two separate monoclonal antibodies (Lee et al., 2016; Hu et al., 2017; Jimeno et al., 2019). Antibody-drug conjugates are designed to target cancer cells with potent cytotoxic drugs while minimising damage to surrounding healthy cells and tissues, reducing side effect risk and severity. For example, PF-06650808 is a novel anti-Notch3-auristatin conjugate that has demonstrated manageable toxicity and signs of anti-tumour activity in breast cancer patients. The anti-Notch3 component binds the agent to Notch3-expressing tumour cells, where it is internalised and trafficked to vesicles containing proteolytic enzymes. These enzymes cleave the linker connecting the two components, releasing the auristatin-based payload into the cytoplasm where it induces cell cycle arrest and apoptosis (Rosen et al., 2020).

Recent research has begun investigating whether Notch signalling could be harnessed in immunotherapy-based cancer treatment. Notch is important in lineage determination in the haematopoietic system where it helps to direct the differentiation of CD4+ T helper cells into T_H_1 and T_H_2 subsets. The T_H_1 response is generally considered to be anti-tumorigenic, meaning that if Notch could be very specifically and carefully activated to induce the T_H_1 response, then immune cell anti-cancer activity could be increased (Nowell and Radtke, 2017). For instance, Kondo et al. (2017) generated induced stem cell memory T (iT_SCM_) cells with potent anti-tumour activity from activated CD4+ and CD8+ T cells by co-culture with stromal cells expressing DLL1. In these circumstances, systemically inhibiting Notch signalling would be a hindrance rather than a help.

## Discussion

Over the last 20 years, it has become abundantly clear that Notch signalling plays an important role in both the development of the mammary gland and the aetiology of breast cancer. Within the normal mammary gland, Notch signalling is important in driving multipotent foetal MaSCs into the unipotent luminal progenitor cell fate and in maintaining the progenitor fate through puberty and adult life. In breast cancer, elevated Notch signalling is seen in all cancers but it is particularly associated with TNBC and cancers that show therapy resistance where elevated Notch signalling is associated with poor prognosis.

Given the role Notch signalling plays in lineage commitment within the normal mammary gland as well as in the self-renewal of breast cancer stem cells, it is worth speculating that the majority of breast cancers arise from the unipotent luminal progenitor cells. This is in keeping with several studies looking at the cell-of-origin of breast cancers. Firstly, Molyneux et al. (2010) and Melchor et al. (2014) elegantly demonstrated that the loss of BRCA1 within luminal progenitors in mice leads to the development of basal-like tumours and subsequently, depending on the initiating genetic insult, that luminal-like, basal-like and normal-like tumours can all arise from luminal progenitors. Secondly, the cell-of-origin for the luminal-like tumours that arise in MMTV-PyMT and MMTV-Neu mice and the basal-like tumours that arise in Etv6-NTRK3 mice were all found to be luminal progenitor cells (Tao et al., 2015). Lastly, transforming luminal cells isolated from normal human tissue by virally introducing a variety of oncogenes leads to the formation of both ER+ve and ER-ve breast cancers when the cells are transplanted into immunocompromised humanised mice (Proia et al., 2011; Keller et al., 2012). Given these observations, it would be interesting to know whether specifically activating Notch signalling within different cells of the luminal lineage is enough to convert the cells into a progenitor-like fate and initiate tumorigenesis.

It is also interesting to note that mature luminal cells and luminal progenitor cells are arranged in mosaic patterns within the mature mammary ductal epithelium similar to those generated by mathematical modelling of Notch signalling (Hadjivasiliou et al., 2016; Bocci et al., 2020; Dawson and Visvader, 2021). These patterns are particularly associated with lateral induction by Notch/JAG signalling which fits well with known expression of JAG1 in the mammary ductal epithelium (Raafat et al., 2011; Xu et al., 2012; Bocci et al., 2020). The role of Notch/JAG signalling within the pattern of mature luminal cells and luminal progenitor cells could be addressed by disrupting signalling specifically within the mature mammary gland.

The involvement of Notch signalling in resistance to all types of breast cancer treatment, including chemotherapy, radiotherapy, endocrine and HER2-targeting therapies, means that Notch inhibition could be valuable in a broad range of patient groups. However, to date it has proven difficult to target Notch signalling in patients. Pan Notch inhibitors have led to unacceptable side effects, whilst we have most likely failed to stratify patients appropriately for homologue-specific Notch inhibitors to be successful. However stratification to signalling through individual Notch proteins may make identifying appropriate patients for therapy too complex. An alternative approach could be to target one of the signalling events downstream of all Notch homologues. Notch activates numerous pro-tumorigenic signalling pathways, some of which have pre-existing inhibitors prime for re-appropriation into breast cancer therapy. Ideally, a pathway would be targeted that has roles in multiple hallmarks of breast cancer, but in particular apoptosis given the role Notch signalling has in cell survival and the enrichment of BCSCs observed following therapy.

## Author Contributions

AE and KB contributed to the systematic review of the literature required for this article, and the writing and editing of the final text. Both authors contributed to the article and approved the submitted version.

## Conflict of Interest

The authors declare that the research was conducted in the absence of any commercial or financial relationships that could be construed as a potential conflict of interest.
